# How a patient-led advocacy organization supports the road to diagnosis and treatment of creatine transporter deficiency

**DOI:** 10.3389/fnins.2025.1548182

**Published:** 2025-02-25

**Authors:** Heidi Wallis, Sangeetha Iyer, Emily K. Reinhardt

**Affiliations:** Association for Creatine Deficiencies, Carlsbad, CA, United States

**Keywords:** creatine transporter deficiency, guanidinoacetate methyltransferase deficiency, l-arginine:glycine amidinotransferase deficiency, patient advocacy, intellectual disability, drug development, newborn screening, X-linked

## Abstract

The current era of drug development has evolved significantly. Patient advocacy organizations are moving beyond simply supporting community members and are taking the reins to improve the speed of diagnoses, initiate therapeutic discoveries, and lay the groundwork to ensure successful clinical trials. The Association for Creatine Deficiencies (ACD) is an international parent-led patient advocacy organization focused on the three ultra-rare neurodevelopmental monogenic disorders resulting in Cerebral Creatine Deficiency Syndromes (CCDS). These include X-linked creatine transporter deficiency (CTD), guanidinoacetate methyltransferase (GAMT) deficiency, and l-arginine:glycine amidinotransferase (AGAT) deficiency. While each is rare in its own right, the unified CCDS community is effectively advancing the field of CCDS with each disorder benefiting from progress made in the other two disease areas. ACD collaborators include caregivers, academic researchers, clinicians, industry partners, and policymakers. Since its founding in 2012, the organization has evolved and achieved significant milestones. These include advancements in disease diagnosis, investments in various therapeutic modalities, creation of a collaborative research community, a unified patient community contributing essential patient data, and repositories of patient-derived specimens. The initiatives of ACD are intended to create the earliest diagnosis possible through newborn screening, to have an effective treatment, and to make disease management strategies available to all members of the CCDS community, including those diagnosed at later stages and experiencing greater effects of the diseases.

## Introduction

The X-linked creatine transporter deficiency (CTD) is one of three ultra-rare genetic Cerebral Creatine Deficiency Syndromes (CCDS). Encoded by the *SLC6A8* gene, the creatine transporter is responsible for shuttling creatine into cells throughout the body, supporting the adenosine triphosphate (ATP) cycle important to cellular metabolism (Fernandes-Pires and Braissant, 2022). Mutations of the *SLC6A8* gene result in impaired ability to transport creatine into the cells of CTD patients. Reduced creatine typically leads to profound developmental delays and lifelong impacts (Stockler-Ipsiroglu and Van Karnebeek, 2014; Mulik and Mercimek-Andrews, 2023). It is estimated that approximately 2.6% of individuals with a neurodevelopmental disorder have CTD (Bahl et al., 2020). Two additional CCDS are relevant to CTD and important to the scientific understanding of creatine deficiency. Guanidinoacetate methyltransferase (GAMT) deficiency and l-arginine:glycine amidinotransferase (AGAT) deficiency are autosomal recessive creatine synthesis disorders that result in CCDS. There are currently no approved treatments available for CTD, but with early diagnosis, GAMT and AGAT deficiencies are amenable to intervention by way of oral creatine supplementation (Schulze and Battini, 2007).

The Association for Creatine Deficiencies (ACD) was established by parents of children afflicted with CCDS in 2012. With a simple initial goal of building community amongst families with shared experiences via a small support group, in the past 12 years, ACD has expanded its impact significantly. ACD’s focus has grown toward providing patient, family, and public education, advocating for early intervention through newborn screening, and promoting and funding medical research. ACD’s strategy is built on three pillars: (1) discovery research, (2) diagnostic advancement, and (3) disease understanding. ACD has had many successes such as advocating for GAMT deficiency to be added to the United States Recommended Uniform Screening Panel (RUSP), creating its fully-owned patient registry, playing a role in the first clinical trial for CTD, and initiating more than 10 research programs with a strong focus on patient-relevant mutations and biosamples. In this article, we will describe the roadmap that ACD developed to achieve its mission, the successes thus far, and the challenges that lie ahead.

## Discovery research

Therapeutic development is typically preceded by a robust understanding of disease pathology built through successive studies on cellular and animal models of the disease. However, attracting research into the basic science of diseases as rare as CTD is challenging for a few reasons - lack of awareness/education about the disorder, perceived limited impact of transformative discoveries because they may be applicable only to a small cohort of patients, and lack of systematic tools such as antibodies, cell lines, and assays that enable early research.

ACD uses grassroots fundraising, grant writing, and private endowments to build a diverse research portfolio, accepting less common and more risky endeavors, and initiating new approaches. Research in preclinical models of CTD has shown that disease correction requires delivery of creatine to the brain (Udobi et al., 2018). Delivery of therapeutics to the brain, whether small molecules, biologics, non-biological complexed drugs (e.g., nanoparticles) or gene therapies, is extremely challenging. For this reason, CTD research must be focused on the pursuit of both tried and tested as well as novel technologies to enable multiple “shots on goal.”

To address these challenges, ACD has taken a three pronged approach: (1) initiating a fellowship program to attract young researchers to the field of CTD, while prioritizing studies on patient mutations; (2) partnering with several external contract research organizations and core facilities to generate proof of concept data around high throughput assays and enabling reagents such as tool antibodies; (3) organizing an annual scientific symposium with an alternating virtual and in-person presence enabling researchers and patient families from all over the world to participate.

Since 2021, ACD has awarded 15 fellowships to a mix of early career researchers and clinicians. To date, at least half of these researchers who were funded by this mechanism have gone on to generate results that served as foundational data for additional grants. These data have also been presented at the annual conferences organized by ACD. In the past 5 years, attendance at these conferences has increased at both virtual and in-person meetings. The in-person convenings have the additional benefit of researchers meeting patients in person and developing a better understanding of the disease impacts. They also encourage collaborations between researchers with overlapping competencies. In total, ACD-funded CTD research has enabled the discovery of pharmacochaperoning drug candidates (unpublished work), *SLC6A8* gene therapy (Fernandes-Pires et al., 2024; Montani et al., 2024), and organoid research (Broca-Brisson et al., 2023).

## Diagnostic advancement

**FIGURE 1:**
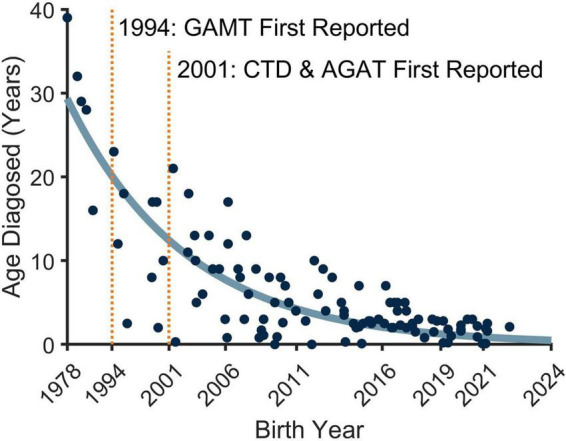
Relationship between patient birth year and age of Cerebral Creatine Deficiency Syndromes (CCDS) diagnosis. Improved access to genetic testing and increased newborn screening of guanidinoacetate methyltransferase (GAMT) deficiency has resulted in CCDS patients (*n* = 111) being diagnosed earlier in their lives, permitting greater opportunity for earlier treatment. The curved line is a two-parameter, exponential descriptive trend line, highlighting the increasing likelihood of detecting a CCDS in younger children.

CTD was first reported in 2001. In the more than 20 years since its discovery, improved access to genetic testing has shortened the time to diagnosis, as it has for other ultra-rare genetic disorders. ACD’s analyses revealed that CCDS patients born during or after 2019 were diagnosed on average at 1.6 years of age. For those born before 2019, patients were diagnosed on average at 7.1 years of age (Association for Creatine Deficiencies, 2024a; Figure 1). While gains have been made in early diagnosis of CCDS, there is still a lack of disease awareness and proactive testing. Children with CTD often display missed milestones, developmental delays, moderate to severe autism, behavior disorders, and seizures. As an X-linked disorder, males often present with more impaired development than females, although this is not always the case (Curie et al., 2024).

ACD envisions an even faster path to diagnosis of CTD with the ultimate goal being diagnosis at birth paired with treatment to mitigate the neurological impacts of CTD. GAMT deficiency has an effective treatment if diagnosed at birth, facilitated by a proven dried blood spot assay for detection of elevated guanidinoacetate in infants. For this reason, ACD petitioned for the addition of GAMT deficiency to the United States RUSP from 2016 to 2021, asking that the federal government recommend to all 50 states that GAMT be included in the routine heel prick screenings nearly all infants receive at birth. GAMT was added to the RUSP in 2023. Advocacy for GAMT newborn screening is underway with 7 states currently screening and 14 more that have declared plans for implementation in the near future (Association for Creatine Deficiencies, 2024b). The RUSP may serve as a model for other nations developing or updating their newborn screening protocols. Shortly after GAMT was added to the RUSP, universal screening in Australia was approved and Alberta, Canada, and Newfoundland and Labrador, Canada, began screening. ACD aims to follow the same playbook for CTD as it did for GAMT: to advocate for a diagnosis at birth. Currently, ACD is collaborating with leading experts in the field of metabolomic and biochemical diagnostics to understand what analytes may identify CTD and how such an assay might be implemented in the current newborn screening system. Early results indicate that creatine levels, already collected as part of GAMT screening, may be the key (Pasquali, 2024). In this case, CTD, though currently lacking a treatment required for RUSP inclusion, may be considered as a secondary finding and reported to the patient’s caregivers and clinician.

A new form of newborn screening is making great advancements toward adoption as an additional tool for identifying diseases at birth - genetic newborn screening (gNBS). gNBS screening panels include a larger number of disorders than traditional panels, thus CTD is more likely to be added to a gNBS panel. One drawback to a genetic screen, versus a metabolite screen, is the need for an understanding of the mutations that cause disease. Over 200 mutations have been identified as pathogenic or likely pathogenic in *SLC6A8* with additional variants being classified as pathogenic regularly (Goldstein et al., 2024a). There is support for gNBS studies to focus on targeted screening of pathogenic and likely pathogenic variants, leaving variants of uncertain significance (VUSs) unreported (Downie et al., 2021). In this situation, a disease-causing VUS would screen negative, resulting in a patient with CTD not receiving the earliest possible diagnosis. ACD has embarked on an effort to reclassify VUSs as pathogenic based on patient-reported data from ACD’s patient registry to ensure the most robust gNBS for CTD possible (Goldstein et al., 2024b; Thomas-Wilson, 2024).

## Disease understanding

### Natural history data

**FIGURE 2:**
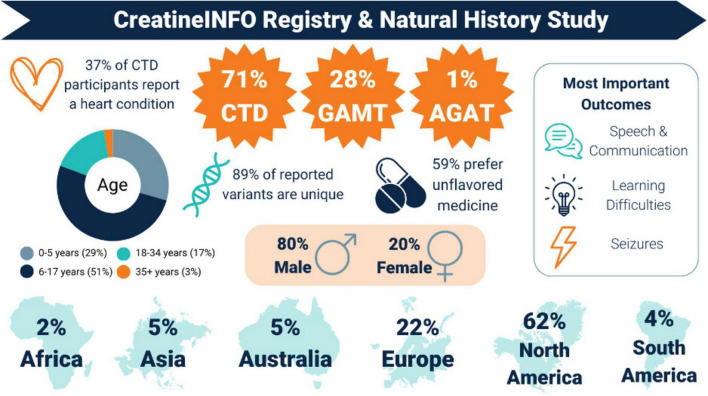
CreatineINFO Registry Data Infographic. This infographic provides a snapshot of the varied topics captured in registry surveys and is an example of how registry data are shared with the Cerebral Creatine Deficiency Syndromes (CCDS) community. The reported percentages (%) are based on the number of responses to a particular registry survey, not based on the total number of participants enrolled in the registry.

Understanding the natural progression of each of the three CCDS is crucial for guiding researchers in developing treatments and helping families plan for their child’s future. As an ultra-rare disease community, there is also power in bringing individual CCDS experiences together to identify patient needs. Toward these goals, ACD launched its own patient- and caregiver-reported registry in 2021 - the CreatineINFO Patient Registry and Natural History Study (Association for Creatine Deficiencies, 2024a). The registry is sponsored and managed by ACD, hosted on the National Organization for Rare Disorders^®^ (NORD^®^) IAMRARE^®^ registry platform, and has Institutional Review Board (IRB) approval. The registry is hosted on a secure platform, complying with United States health information privacy laws and General Data Protection Regulation (GDPR) requirements, thus supporting global participation and representation of CCDS patients and families. ACD’s registry board, composed of caregivers, researchers, and clinicians, serves to (a) identify registry goals and direction, (b) regulate user and data access, and (c) ensure patient relevance. ACD’s registry coordinator collaborates with caregivers, researchers, clinicians, and industry partners to review existing, validated surveys as well as develop new registry surveys designed to collect data and insights (Figure 2). Caregiver feedback is a critical part of the survey development process to ensure that survey questions resonate with participants in both content and language. ACD owns the data collected in its registry, putting them in a unique position to ensure data is accessible. Data ownership has also unlocked a partnership model for key collaborators to request existing registry data and/or deploy custom surveys, in which for-profit industry data requests may require financial registry support. The registry mitigates the “silo-effect” often found in disease research by offering CCDS patients and caregivers a centralized platform for reporting their experiences accurately. This reduces participant burden by limiting requests for their personal data and drives patient-centered research among scientists and industry partners. Currently, over 220 CCDS participants are enrolled in the registry (Association for Creatine Deficiencies, 2024a), with recruitment and retention efforts ongoing. ACD has successfully used social media, webinars, and other community outreach methods to educate CCDS families about the importance of participating in the registry and to share registry findings with the CCDS community.

### Patient experience and priorities

CCDS research is ongoing, and many therapeutics are being explored. ACD recognizes the importance of the patient community to inform research and ensure patient-relevant treatments and clinical trials. CCDS are ultra-rare with very few patients represented in any given clinic. For this reason, it is important to unite patients and caregivers internationally to best inform research. Additionally, the broadest patient representation will ensure patient-centered drug trial design. Historically, patients have not had a primary role in the focus of research or the structure of trials, and ACD aims to change this.

A virtual Externally-Led Patient-Focused Drug Development (EL-PFDD) meeting, scheduled with United States Food and Drug Administration (FDA) approval, was planned and hosted by ACD on 24 January 2023. The meeting was attended by 248 individuals, including one individual living with CTD, parents and caregivers, extended family members, friends, government representatives, healthcare providers, industry representatives, scientists, and non-profit representatives. The meeting included testimonials from key opinion leaders and pre-recorded testimonials from 12 caregivers. Another 10 caregivers participated in live discussion starters with calls from audience members around the world. All attendees were invited to respond to online polling to capture feedback from the widest audience possible. The meeting was divided into two main topics: “Living with CCDS - Symptoms and Daily Impacts” and “Current and Future Approaches to CCDS Treatment.” The findings from this meeting are captured in a comprehensive publication titled “CCDS Voice of the Patient Report” including a summary of important findings identified (Wallis et al., 2023). This public resource is accessible to anyone seeking to develop drugs for CCDS, gives them a headstart in patient-centered development efforts, and serves as a voice for the many CCDS patients that lack the expressive communication to speak for themselves.

### Core outcome set

The selection of consistent outcome measures across future clinical trials is an important consideration in ensuring such trials are patient-centered, comparable, and successful. ACD led an international patient engagement and core outcome set (COS) development project titled “Parents Advancing REsearch NeTworkS (PAReNts)” from 2022 to 2023, which was funded by the Patient-Centered Outcomes Research Institute^®^ (PCORI^®^). A cohort of caregiver participants were at the center of this multi-phased project; 25 caregivers were recruited from the international CCDS community to participate in monthly workshops with a focus on caregiver research engagement training. A literature review was conducted to identify potential outcomes relevant to GAMT and CTD. Through a community wide Delphi survey including caregivers, patients, health professionals, and researchers, the pool of potential outcomes was narrowed. Ultimately a consensus workshop was held with equal representation from the caregiver and health professional communities, and eight outcomes were identified as the COS for CTD and GAMT deficiency (Nasseri Moghaddam et al., 2024; COMET Initiative, 2024). This resource has two strong benefits - to provide the design of clinical trials with thoughtful caregiver input as required by regulators, and to ensure the quality of outcome selection in future trials.

### Considerations for the selection of outcome measurement tools

Upon completion of the COS project, participating caregivers and subject matter experts agreed that more work was needed to ensure the proper measurement of the COS in eventual trials. There is a high level of heterogeneity amongst CTD and GAMT patients, especially between female and male CTD patients and between GAMT patients diagnosed at different ages. Any means of measuring the eight outcomes would need to be sensitive enough for small changes as well as broad enough in scope to capture very different changes in this diverse population. A second PCORI^®^ grant was secured and the Parents Advancing REsearch NeTworkS: Expand Community, Tools & Engagement (PAReNts 2.0: ExCiTE) began in September 2024. This project once again begins with a cohort of caregivers to train and direct the priorities of the project throughout the grant. Caregivers will engage in a focus group type “exploratory jam” to discover the small but meaningful changes they anticipate in each of the eight core outcomes measured within a successful clinical trial. The research team will conduct a literature review to identify tools used in other clinical trials for similar neurodevelopmental disorders as potential tools. Subject-matter experts will discuss the literature review and focus group data to provide an additional view on feasibility and access of tools. The project will conclude with caregivers, patients, subject-matter experts, health professionals, industry, policymakers, and researchers at an in-person alignment meeting for discussion and further revisions. The results of this meeting, and the entirety of the project, will be documented as “Considerations for the Selection of Outcome Measurement Tools” and distributed widely.

### Industry engagement

ACD is focused on enriching the general understanding of patient and caregiver challenges with key collaborators, including industry partners. In this vein, providing access to community insights and collaborating on community education efforts is especially meaningful in advancing clinical trial recruitment and participation readiness. In the past 12 years, several industry partners have collaborated with ACD while developing therapies. CTD has gained the most industry partner interest because (1) it is the most common CCDS and (2) it lacks any current therapeutic alternatives. By collaborating with ACD, our industry partners have leveraged the wealth of insights and data collected by ACD to develop better therapeutics. ACD engages with industry partners through its Corporate Alliance for Registry and Education (C.A.R.E.) consortium (Association for Creatine Deficiencies, 2024c). Through the consortium, industry partners are encouraged to share their goals, progress, and any challenges encountered in developing CCDS therapies. In return, ACD provides access to insights such as patient mutations, biospecimens, patient registry data, patient-centered clinical outcomes of interest, connections to key researchers, and models necessary to drive research forward. In recent years, ACD’s registry data has proved to be a valued asset for recruitment and survey data insights for industry partners as well as clinical investigators. ACD also provides a platform for outreach and education to our community members as part of the C.A.R.E. consortium benefits and collaborates with C.A.R.E. partners in strategies for educating the CCDS community toward clinical trial readiness.

## Discussion

As is evidenced from this perspective, patient organizations are a key driver of collaboration and change in a community consisting of caregivers, academics, clinicians, policy makers, and industry partners. Patient organizations play an important role in organizing efforts at the grassroots level to advocate for better representation, outcomes, and treatments. Taking a step beyond the traditional role of raising awareness and community support, ACD is building a diverse research portfolio that will enable multiple paths toward potential treatments for our community. ACD is prioritizing the collection of community data to inform patient-centered representation in research and clinical outcomes. These data-driven insights enable an evidence-based approach to strategic research planning, bolstering credibility with partners, and advocating for advancement of treatments for the CCDS community.

As representatives of an ultra-rare neurodevelopmental disease community, ACD offers critical insights and resources for industry partners who are invested in therapies targeting the CCDS community. While industry partners may need to make pivots to support their financial and strategic goals, patient organizations remain committed to their patient community in spite of changing industry landscapes. ACD is actively engaged in building a framework along discovery research, diagnostic advancement, and patient-centered disease understanding to ensure community readiness for future therapies. ACD’s roadmap for successfully advocating for the CCDS community has evolved to its current state over the past 12 years and has been inspired by the collective experiences of numerous patient advocacy groups. Indeed, ACD will continue to work towards the vision of effective treatments and diagnosis through newborn screening for all CCDS patients to ensure the best possible outcomes for this underserved community.

## Data Availability

The data analyzed in this study are subject to the following licenses/restrictions: Datasets represented in this article are collected as part of ACD’s registry and may not be replicated without written permission from ACD. Requests to access these datasets should be directed to registry@creatineinfo.org.
